# Improving the Physical and Oxidative Stability of Emulsions Using Mixed Emulsifiers: Casein-Octenyl Succinic Anhydride Modified Starch Combinations

**DOI:** 10.3390/nano9071018

**Published:** 2019-07-16

**Authors:** Liu Yang, Xiaoli Qin, Jianquan Kan, Xiong Liu, Jinfeng Zhong

**Affiliations:** 1 College of Food Science, Southwest University, Chongqing 400700, China; 2 Chongqing Key Laboratory of Soft-Matter Material Chemistry and Function Manufacturing, Southwest University, Chongqing 400700, China

**Keywords:** oil-in-water nanoemulsion, protein, modified starch, physical stability, oxidative stability

## Abstract

This study aims to investigate the influence of casein and octenyl succinic anhydride modified starch (OSAS) combinations on the physical and oxidative stability of fish oil-in-water emulsions. The interaction between casein and OSAS was manifested in changes in protein structure and hydrogen-bonding interaction. Casein–OSAS combinations could effectively inhibit droplet aggregation at pH 4 and attenuate droplet growth at a high CaCl_2_ concentration of 0.2 mol/L, compared with casein as an emulsifier. Nanoemulsions stabilized by casein–OSAS combinations or casein showed better oxidative stability compared with OSAS-stabilized emulsions. Therefore, casein–OSAS combinations can improve some physical properties of protein-based emulsions and oxidative stability of modified starch-based emulsions, suggesting protein-modified starch combinations are more promising in the emulsion-based food industry compared to each of the two emulsifiers alone.

## 1. Introduction

Oil-in-water emulsions have potential applications in the food industry for protecting lipophilic bioactive compounds from chemical degradation, improving dispersibility, and enhancing bioavailability [1,2]. Proteins and polysaccharides are two common types of emulsifiers frequently used to form and stabilize oil-in-water emulsions [3,4,5]. However, either proteins or polysaccharides as individual emulsifiers have their own advantages and disadvantages. For example, proteins usually adsorb quickly to the interface and coat the oil droplets formed during homogenization to prevent them from aggregating again [4]. However, protein-stabilized emulsions were shown to be unstable at pH values close to the isoelectric point of the protein and at high ionic strengths [6,7,8]. Polysaccharides can produce emulsions with excellent stability, but the levels of polysaccharides required to form stable emulsions are generally much higher than those of globular proteins [9,10]. Such disadvantages of emulsions coated by the individual type of emulsifier may limit their wide applications in food systems and processing. Therefore, there is considerable interest nowadays in using protein–polysaccharide combinations to improve the stability of emulsions coated by an individual emulsifier [11,12].

Because of rapid adsorption of proteins and steric repulsion and viscosity enhancement of polysaccharides, the combination of proteins and polysaccharides to stabilize emulsions has achieved favorable effects. Protein–polysaccharide complexes that improve the emulsion stability can be formed by covalent bond and non-covalent association [13,14,15,16]. Protein–polysaccharide complexes formed by non-covalent association are more common in improving the physical stability of emulsions, because the conditions for the formation of non-covalent association are easier to achieve [17]. For example, Li et al. found that when 1% of gum Arabic was added at pH 8, the rapeseed protein isolate-stabilized emulsion obtained the lowest centrifugal precipitation rate, the maximum absolute value of ζ-potential and smaller particle size, showing enhanced physical stability [18]. However, only a few studies have focused on both the physical and oxidative stability of emulsions using protein–polysaccharide combinations as emulsifiers [19,20], which limits the exploration of candidate protein–polysaccharide combinations as potential emulsifiers in industries.

Octenyl succinic anhydride modified starch (OSAS) is derived from the modification of starch molecules with hydrophobic octenyl succinic anhydride and has amphiphilic properties. This modified starch with advantages of low-cost and abundance has been used as emulsifying and stabilizing agents [21,22]. OSAS has been demonstrated to stabilize emulsions mainly through steric repulsion, making them less impacted by pH and ionic strength [23]. Therefore, OSAS has the potential to improve the physical stability of emulsions when used as one of the mixed emulsifiers. As previously reported, lecithin-coated emulsion was stable at ≤100 mmol/L NaCl [24], while emulsion prepared with OSAS–phosphatidylcholine combinations was stable at higher NaCl concentrations (≤800 mmol/L) [25]. Although modified starch gives good physical stability to emulsions, only a few reports are available on the effect of modified starch on the oxidative stability of emulsions [26]. In particular, the research on the oxidative stability of emulsions using the combination of OSAS and protein as emulsifiers is limited. The effects of protein and OSAS as emulsifier combinations on the physical and oxidative stability of emulsions are unclear.

Therefore, casein was selected as a protein model to study the formation and stability of nanoemulsions prepared with casein–OSAS combinations. First, the effect of major variables on the formation and stabilization of nanoemulsions was evaluated. Second, the influences of environmental variables (i.e., pH, ionic strength, and storage conditions) on the physical and oxidative stability of the nanoemulsion prepared with casein–OSAS combinations and the emulsion prepared with each of the two emulsifiers alone were compared.

## 2. Materials and Methods

### 2.1. Materials

Casein was supplied by the Hefei Bomei Biotechnology Co., Ltd. (Hefei, China). Octenyl succinic anhydride modified starch (Purity Gum 2000) was purchased from the National Starch and Chemicals (Shanghai, China). Fish oil was kindly donated by Sinomega Biotech Engineering Co., Ltd. (Zhoushan, China). Medium-chain triglycerides (MCTs) were purchased from the Shanghai Yiji Industrial Co., Ltd. (Shanghai, China). *γ*-Oryzanol was purchased from Dalian Meilun Biotech Co., Ltd. (Dalian, China). KBr (spectral purity) was purchased from Tianjin Guangfu Fine Chemical Research Institute (Tianjin, China). The polystyrene latex particles were purchased from Tianjin Saierqun Technology Co., Ltd. (Tianjin, China). All the other reagents used were of analytic grade.

### 2.2. Emulsion Preparation

Initially, casein dispersions were prepared by adding casein powder into ultrapure water and then stirring at 500 rpm at 30 °C for 40 min. After adding sodium azide solution (0.02% final concentration) for inhibiting microbial growth, the casein dispersions were kept at 4 °C for 5 h to ensure dispersion and hydration. The aqueous phase was prepared by adding OSAS into the casein dispersions and then stirring at 500 rpm at 30 °C for 40 min. The oil phase was prepared by adding *γ*-oryzanol (1% by the total weight of oils) into the oils and then stirring at 500 rpm at 50 °C for 30 min. *γ*-Oryzanol was used as a natural antioxidant, and the amount of *γ*-oryzanol added was according to our previous study [27]. Then, the aqueous phase was added to the oil phase with stirring at 700 rpm at 30 °C. After an additional 20 min stirring, the 70 mL mixture in a 150 mL beaker was homogenized to produce a coarse emulsion using an Ultra Turrax T18 homogenizer (IKA, Staufen, Germany) operating at 20,000 rpm for 3 min. The obtained coarse emulsion was then homogenized by an ultrasound processor with a 20 mm diameter probe (JY98-IIIDN, Ningbo Scientz Biotechnology Co., Ltd., Zhejiang, China). The probe was immersed 25 mm below the liquid surface. The frequency was 20 kHz and pulse duration was set as on-time 5 s and off-time 2 s. The sample temperature was controlled ≤50 °C using an ice-water bath during the emulsion preparation.

To obtain nanoemulsions with excellent stability, the effects of formulation and process parameters on the particle size and ζ-potential of emulsions were investigated by single-factor tests according to Table 1. To compare the effects of emulsifiers (casein, OSAS, and their combination in a casein/OSAS weight ratio of 8:2) on physicochemical properties of emulsions, another set of emulsions was prepared under the optimized conditions (1% fish oil phase, 7% emulsifier, 92% ultrapure water and ultrasound for 22 min at 360 W).

### 2.3. Physicochemical Stability of Emulsions Evaluation

The physical stability of emulsions under various environmental stresses was evaluated by determining changes in particle size and ζ-potential. The influence of pH was evaluated by adjusting the pH of emulsions to different pH values (2–9) using 1 mol/L HCl or NaOH solution. To examine the influence of ionic strength, a series of emulsions with different concentrations of NaCl (0–1.0 mol/L) or CaCl_2_ (0–0.2 mol/L) were prepared by adding a small amount of NaCl or CaCl_2_ solution. All the treated emulsions were stored for 40 h prior to particle size and ζ-potential measurements.

The oxidative stability tests were performed by determining changes in primary and secondary lipid oxidation products during storage of emulsions at 4 and 25 °C for 6 weeks. Emulsions withdrawn at different times were stored at −80 °C until further analysis. The peroxide value representing primary lipid oxidation products was determined according to the procedure described by Zhong, Liu, Wang, Qin, and Li [28]. The *p*-anisidine value representing secondary lipid oxidation products was determined according to the standard method [29].

### 2.4. Particle Size, Polydispersity Index (PDI), and ζ-Potential Measurements

The particle size, PDI and ζ-potential of emulsions were determined using a Malvern Zetasizer Nano ZS90 instrument (Malvern Instruments, Worcestershire, UK). To avoid multiple scattering effects, all the emulsion samples were diluted 1000 times with ultrapure water. The viscosity and refractive index of water were 0.8872 cP and 1.330, respectively. The measurement angle was set at 90°. Each individual measurement was obtained as an average of 13 runs. All the measurements were performed at least in duplicate at 25 °C. The average particle size is expressed as the z-average diameter (i.e., mean hydrodynamic diameter).

### 2.5. Fourier Transform Infrared (FTIR) Spectroscopy Measurement

The structure and interaction of casein and OSAS were measured using an FTIR spectrometer (Spectrum 100, PerkinElmer, Waltham, MA, USA). The aqueous phases used for emulsions preparation were freeze-dried to ensure no water existed in the samples (casein, OSAS, and the combination of casein and OSAS in a weight ratio of 8:2). Each sample was pressed into a transparent sheet by a tableting machine with KBr in a weight ratio of 1:100. The scanning range was 4000–400 cm^−1^, the spectral resolution was 4 cm^−1^, and the number of scans was 32 times.

### 2.6. Interfacial Layer Thickness Measurement

Interfacial layer thickness was measured to evaluate the relationship between the z-average diameter of emulsions and the interfacial layer thickness. The layer thickness of adsorbed casein, OSAS or the combination of casein and OSAS in a weight ratio of 8:2 onto the latex surface was determined according to the method described by Liu, Yadav, and Yin [30] with slight modification. The sample solution (casein, OSAS or the combination of casein and OSAS in a weight ratio of 8:2 solution at 7% concentration) and the polystyrene latex aqueous suspension (1 mg/mL) were mixed in a volume ratio of 1:1 and then allowed to stand for 2 h. The latex-sample solution mixture was measured according to the method in Section 2.4. The hydrodynamic diameter of the polystyrene latex particles was 171.6 ± 3.2 nm. The interfacial layer thickness was calculated using the following equation:

(1) Interfacial layer thickness=(A−N)/2

where *N* is equal to 171.6 nm, and *A* is the hydrodynamic diameter (nm) of particles with adsorbed casein, OSAS or the combination of casein, and OSAS in a weight ratio of 8:2.

### 2.7. Morphology Measurement

The morphological characteristics of emulsions were observed by transmission electron microscopy (TEM, JEOL JEM-1400, JEOL Ltd., Tokyo, Japan) according to the procedure described by Zhong, Wang, and Qin [25].

### 2.8. Tribological Measurement

Tribology is used to evaluate the thin-film properties of materials such as food hydrocolloids and emulsions [31]. Tribological behaviors of emulsions were measured in a ball-on-three-plates system using an MCR 302 Rheometer (Anton Paar, Graz, Austria). During the tests, the normal force acting on the ball was set as 1 N. The sliding speed was increased from 10^−5^ to 1 m/s at 25 °C. The friction coefficient of the emulsion was recorded as a function of the sliding speed.

### 2.9. Statistical Analysis

All the experiments were performed at least in duplicate, and all the measurements were determined at least in duplicate. The results were present as the mean values ± standard deviation. Analysis of variance test was performed using SPSS software (version 14.0 demo; SPSS Inc., Chicago, IL, USA) to express differences between the observed mean values. Significant differences were considered as *p* < 0.05.

## 3. Results and Discussion

### 3.1. Effect of Variables on the Formation and Stabilization of Nanoemulsions

#### 3.1.1. Effect of the Casein/OSAS Weight Ratio

**Figure 1:**
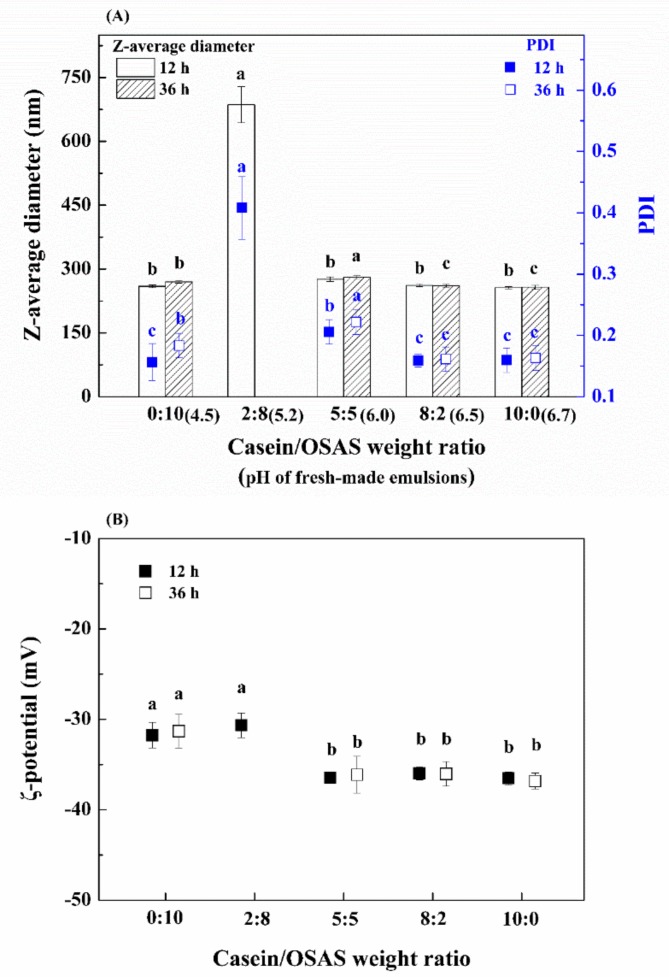

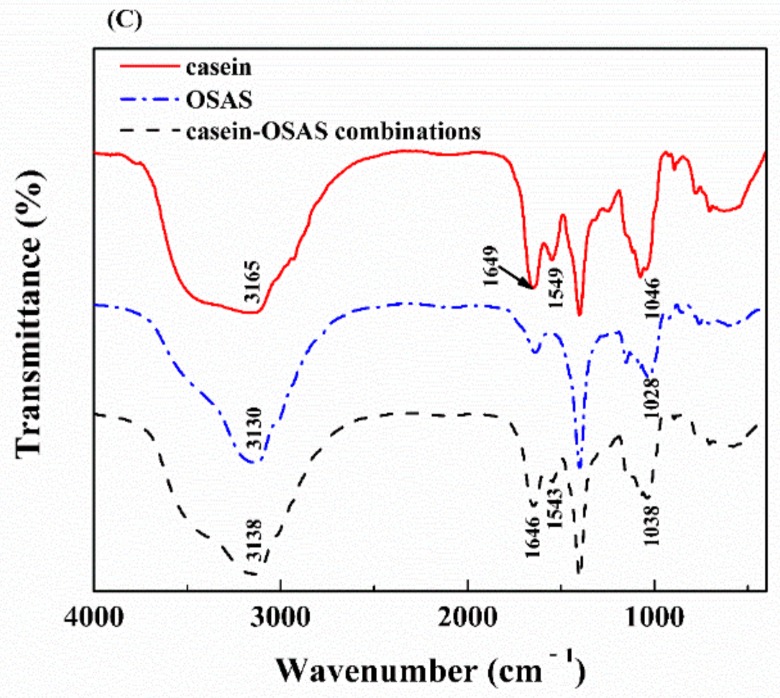
(**A**) Effect of the casein/octenyl succinic anhydride modified starch (OSAS) weight ratio on the z-average diameter and polydispersity index (PDI) of fish oil emulsions. (**B**) Effect of the casein/OSAS weight ratio on the ζ-potential of fish oil emulsions. Different lowercase letters above the same type of columns or the same type of symbols indicate significant difference (*p* < 0.05). (**C**) The FTIR spectra of casein, OSAS, and the combination of casein and OSAS in a weight ratio of 8:2, respectively.

Figure 1A,B shows the influence of the casein/OSAS weight ratio on the physical stability of emulsions. Interestingly, when the casein/OSAS weight ratio was 2:8, an unusually large z-average diameter was observed and the PDI was significantly high (*p* < 0.05) which indicated that the emulsion had a wide particle size distribution. This is because the pH value (5.2) of the emulsion close to the isoelectric point of casein (approximately 4.6) led to electrostatic interactions and low solubility of the protein, thereby decreasing its emulsifying capacity [32]. The z-average diameter (approximately 259 nm at 12 h) of the emulsion prepared with OSAS alone was only slightly larger than that (approximately 256 nm at 12 h) of the emulsion prepared with casein alone. However, a more rapid increase in z-average diameter was observed in the emulsion prepared with OSAS alone during storage for 36 h. This may be because the absolute value of ζ-potential of the emulsion prepared with OSAS alone was significantly lower than that of the emulsion prepared with the combination of casein and OSAS in a weight ratio of 5:5, 8:2, and 10:0, respectively (*p* < 0.05) (Figure 1B). A higher absolute value of the ζ-potential generally leads to more stable emulsions stabilized primarily by electrostatic repulsion [33]. Since the appropriate addition of OSAS had almost the same effect on emulsion stabilization compared with casein, a combination of casein and OSAS in a weight ratio of 8:2 was selected as emulsifier combination for the subsequent experiments.

The structure and interaction between casein and OSAS were examined using FTIR spectroscopy. As shown in Figure 1C, the bands of amide І (1700–1600 cm^−1^) and amide Ⅱ (1550–1500 cm^−1^) are the most prominent characteristic bands in the protein polypeptide chain. The absorption peak positions of amide І (–C = O stretching vibration) and amide Ⅱ (–C–N stretching vibration and –N–H bending vibration) bands were observed at 1649 and 1549 cm^−1^ in casein, respectively. While for the combination of casein and OSAS in a weight ratio of 8:2, they were observed at 1646 and 1543 cm^−1^, respectively. The absorption peak positions of the two bands of the combination of casein and OSAS in a weight ratio of 8:2 were red shifted compared with casein, indicating the changes of the complex condensation of the carbonyl region in the combination of casein and OSAS in a weight ratio of 8:2 [18]. In addition, OSAS showed a prominent peak at 1028 cm^−1^ attributed to the C–OH stretching vibration and casein showed a distinct absorption peak at 1046 cm^−1^. However, the absorption peak appeared at 1038 cm^−1^ for the combination of casein and OSAS in a weight ratio of 8:2. These changes, which could be related to side-chain vibrations of the protein, indicated alteration in protein structure [34]. The band in the range of 3500–3000 cm^−1^ was correlated with O–H stretching vibration and the absorption peak appeared at around 3165, 3138 and 3130 cm^−1^ for casein, the combination of casein and OSAS in a weight ratio of 8:2 and OSAS, respectively. This implied hydrogen bonding was also involved in the interaction between casein and OSAS [18].

#### 3.1.2. Effect of Other Formulation Parameters

**Figure 2:**
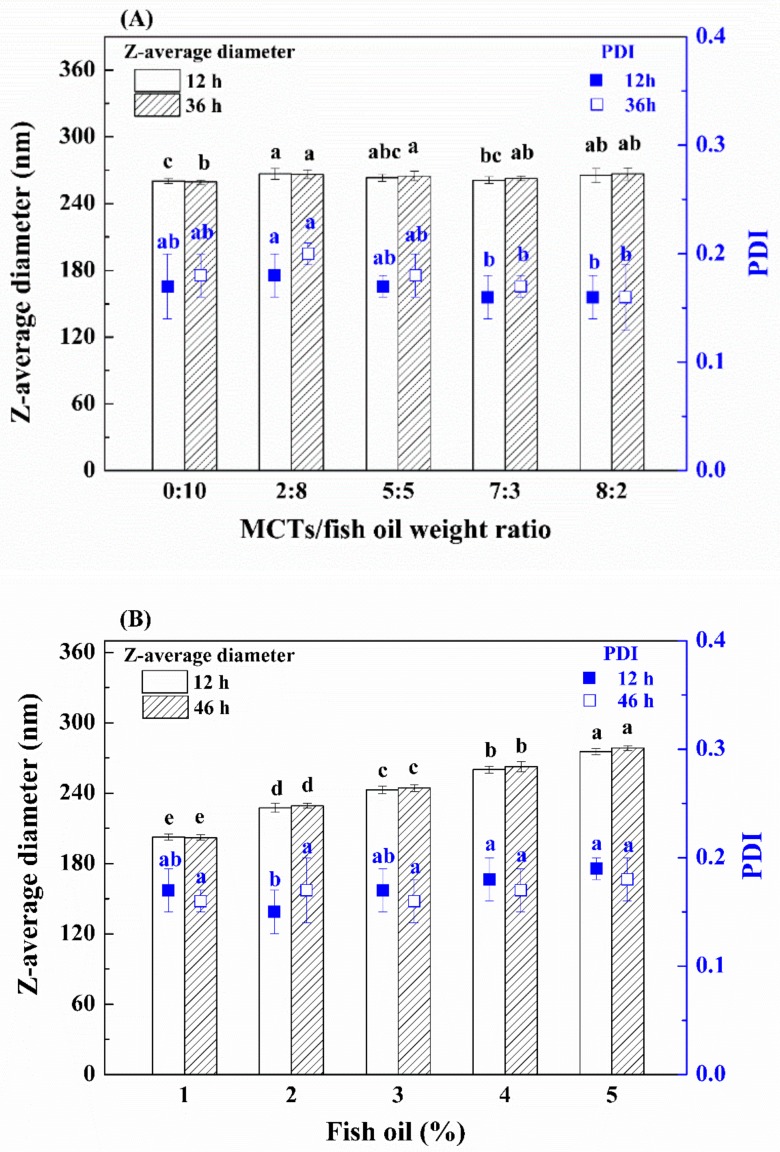
Effect of the composition (**A**) and concentration (**B**) of the oil phase on z-average diameter and PDI of fish oil emulsions. Different lowercase letters above the same type of columns or the same type of symbols indicate significant difference (*p* < 0.05).

Other formulation parameters including the composition and concentration of oils in emulsions were studied. Generally, the type or composition of oils exhibit a different effect on the formation and stabilization of nanoemulsions, which may be related to the different physicochemical properties of oils and the energy required for the breakdown of large-size oil droplets onto nano-size ones [35]. Previous studies have shown that nanoemulsions prepared by low-energy emulsification methods contained much smaller droplets when MCTs rather than long-chain triglycerides were used as the oil phase [36]. In this study, the effect of the composition of the oil phase on the physical property of emulsions prepared by a high-energy emulsification method (i.e., ultrasonic emulsification) was examined. Increasing the level of fish oil in the oil phase led to a slight change (<5.2 nm) in the z-average diameter of emulsions stored for 12 h (Figure 2A), indicating adding MCTs was not favorable for the formation of much smaller emulsion droplets when the ultrasonic homogenizer was used. In addition, the PDI of the emulsion prepared with only fish oil was not significantly different from that of the emulsions containing MCTs (*p* > 0.05) (Figure 2A). In order to increase the concentration of fish oil in emulsions, an oil phase consisting of only fish oil was selected for the rest of the experiments.

Emulsions containing different concentrations of fish oil had similar PDI (Figure 2B). However, an increase in oil phase from 1% to 5% led to a linear increase by 72.9 nm (from 202.5 to 275.4 nm) in the z-average diameter of emulsions stored for 12 h (Figure 2B). Greater changes in z-average diameter of the emulsions containing 3%, 4%, and 5% oil were shown as the storage time proceeded compared with emulsions with lower oil concentrations (i.e., 1% and 2%). On the other hand, there was no whitish layer on the surface of all emulsions at 12 h. However, thin whitish layers were observed on the surface of the emulsions prepared with higher oil concentrations (i.e., 2%, 3%, 4%, and 5%) after 24 h, indicating the emulsions became unstable. There was no visible creaming at the top of the nanoemulsion prepared with 1% oil until 48 h. Therefore, 1% fish oil was chosen for further study.

#### 3.1.3. Effect of Process Parameters

**Figure 3:**
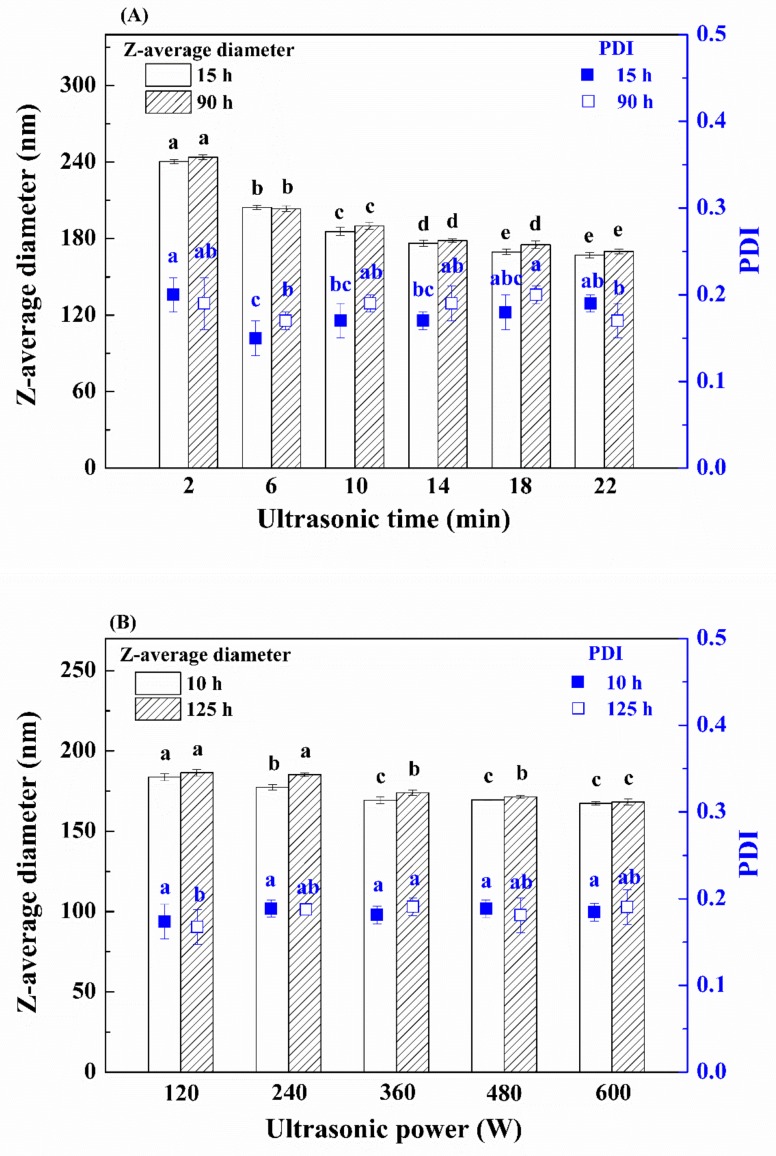
Effect of ultrasonic time (**A**) and ultrasonic power (**B**) on the z-average diameter and PDI of fish oil emulsions. Different lowercase letters above the same type of columns or the same type of symbols indicate significant difference (*p* < 0.05).

Process parameters including ultrasonic time and power also govern the formation and stabilization of nanoemulsions. As shown in Figure 3, all emulsions had low PDI, indicating that they had a narrower particle size distribution. A decrease by approximately 55 nm in the z-average diameter of emulsions was achieved within the first 8 min of ultrasonic time, while a decrease by only 16 nm in z-average diameter was obtained by the next 8 min (Figure 3A). An additional increase in ultrasonic time from 18 to 22 min had no significant effect (*p* > 0.05) on the z-average diameter of nanoemulsions stored within 15 h. However, the z-average diameter seemed to become larger with increasing storage time to 90 h for the nanoemulsion prepared by ultrasonic treatment of 18 min compared with the nanoemulsion prepared by ultrasonic treatment of 22 min. Considering the long-term nanoemulsion stability, the ultrasonic time of 22 min was selected. When ultrasonic power increased from 120 to 360 W, the z-average diameter of nanoemulsions significantly decreased (*p* < 0.05) (Figure 3B). No significant change in z-average diameter (*p* > 0.05) was obtained with increasing ultrasonic power from 360 to 600 W. Therefore, the ultrasonic power of 360 W was chosen, and a stable nanoemulsion with small z-average diameter (approximately 170 nm) was obtained under the optimized conditions.

### 3.2. Comparison of the Physical and Oxidative Stability of Emulsions Prepared with Various Emulsifiers

#### 3.2.1. Particle Size and Morphology

**Figure 4:**
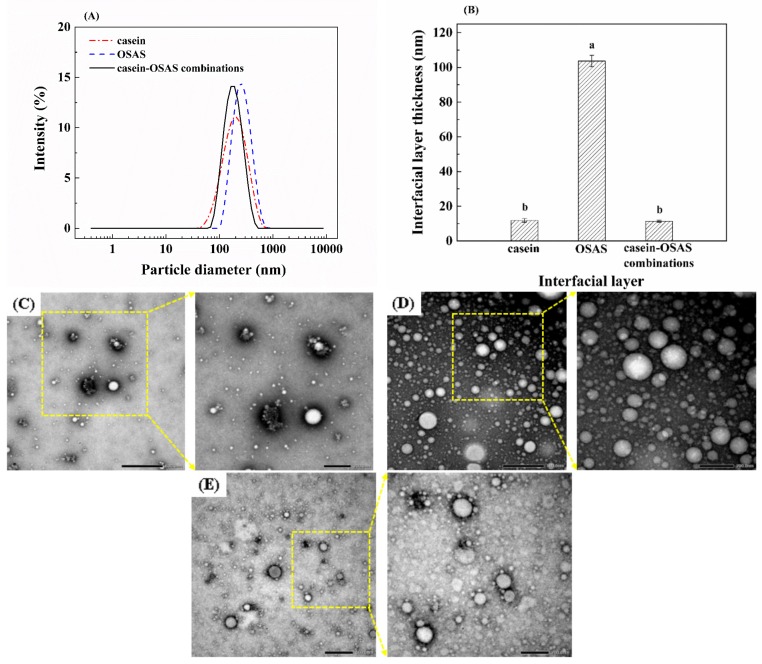
(**A**) Particle size distribution of emulsions stabilized by casein, OSAS, and the combination of casein and OSAS in a weight ratio of 8:2, respectively. (**B**) Interfacial layer thickness formed by casein, OSAS, and the combination of casein and OSAS in a weight ratio of 8:2, respectively. Different lowercase letters above the columns indicate significant difference (*p* < 0.05). (**C**–**E**) TEM images of emulsions prepared with casein, OSAS, and the combination of casein and OSAS in a weight ratio of 8:2, respectively.

The particle size distribution and morphology of emulsions were characterized by dynamic light scattering and TEM, respectively. Nanoemulsions stabilized by casein or the combination of casein and OSAS in a weight ratio of 8:2 had similar and relatively small z-average diameter (approximately 170 nm), while OSAS-stabilized emulsion showed relatively z-average diameter (approximately 236 nm) (Figure 4A). Correspondingly, casein and the combination of casein and OSAS in a weight ratio of 8:2 formed a similar and thin interfacial layer (11–12 nm), while OSAS formed an extremely thick interfacial layer (approximately 104 nm), as shown in Figure 4B. Therefore, there was a positive correlation between the z-average diameter (i.e., mean hydrodynamic diameter) and the interfacial layer thickness, indicating that the interfacial layer thickness is one of the reasons affecting the z-average diameter [15]. In addition, the PDI of three emulsions stabilized by casein, OSAS, and the combination of casein and OSAS in a weight ratio of 8:2 were 0.187, 0.155, and 0.174, respectively, indicating that the particle size distribution of the three emulsions was narrow. As expected, particle sizes measured by TEM (Figure 4C–E) were smaller than those given by dynamic light scattering (Figure 4A). This is because the TEM measurements require an emulsion solution to be in a dry state (i.e., most compact state) by air-drying while the dynamic light scattering measurements give the hydrodynamic diameter of the particles. TEM images showed that the three emulsions (prepared with casein, OSAS, and the combinations of casein and OSAS in a weight ratio of 8:2, respectively) had individual particles (Figure 4C–E), indicating no sign of flocculation was observed in these emulsions.

#### 3.2.2. Influence of pH

**Figure 5:**
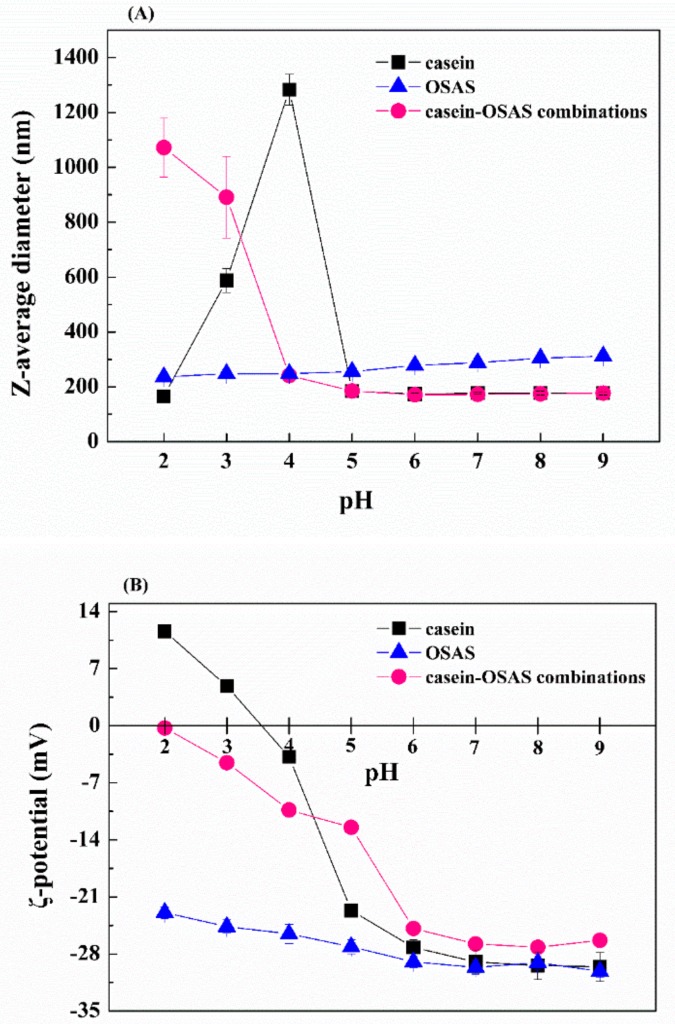
Influence of pH value on the z-average diameter (**A**) and ζ-potential (**B**) of emulsions stabilized by casein, OSAS, and the combination of casein and OSAS in a weight ratio of 8:2, respectively.

The influence of pH on the z-average diameter of the three emulsions (prepared with casein, OSAS, and the combinations of casein and OSAS in a weight ratio of 8:2, respectively) was investigated (Figure 5A). The largest z-average diameter (approximately 1283 nm) of casein-coated nanoemulsion was obtained at pH 4, indicating the aggregation of casein-coated lipid droplets occurred around the isoelectric point of casein due to low electrostatic repulsion (ζ-potential = −3.82 mV, as shown in Figure 5B). Interestingly, the combination of casein and OSAS in a weight ratio of 8:2 could prevent droplet aggregation at pH 4, as seen by a relatively small z-average diameter (approximately 241 nm). This effect can be attributed to two reasons: (1) there would be increased steric repulsion between the droplets due to the adsorbed OSAS interfacial layer, which may have been strong enough to prevent droplet aggregation. Recent studies have demonstrated that OSAS- coated lipid droplets have excellent stability against aggregation at a wide range of pH values due to steric (rather than electrostatic) repulsion [23,37]; (2) there was electrostatic interaction between the casein and the OSAS, which was confirmed by the ζ-potential value measurement. At relatively low pH values (2–4) the ζ-potential values of the nanoemulsion prepared with the combination of casein and OSAS in a weight ratio of 8:2 were lower than those of casein-coated nanoemulsion and higher than those of OSAS-coated emulsion (Figure 5B), presumably the anionic groups (–COO^−^) on the OSAS bound to cationic groups (–NH_3_^+^) on the casein through electrostatic attraction. In summary, the combination of casein and OSAS in a weight ratio of 8:2 provide good protection against droplet aggregation at pH close to the isoelectric point of protein due to a combination of electrostatic and steric stabilization mechanisms.

#### 3.2.3. Influence of Ionic Strength

**Figure 6:**
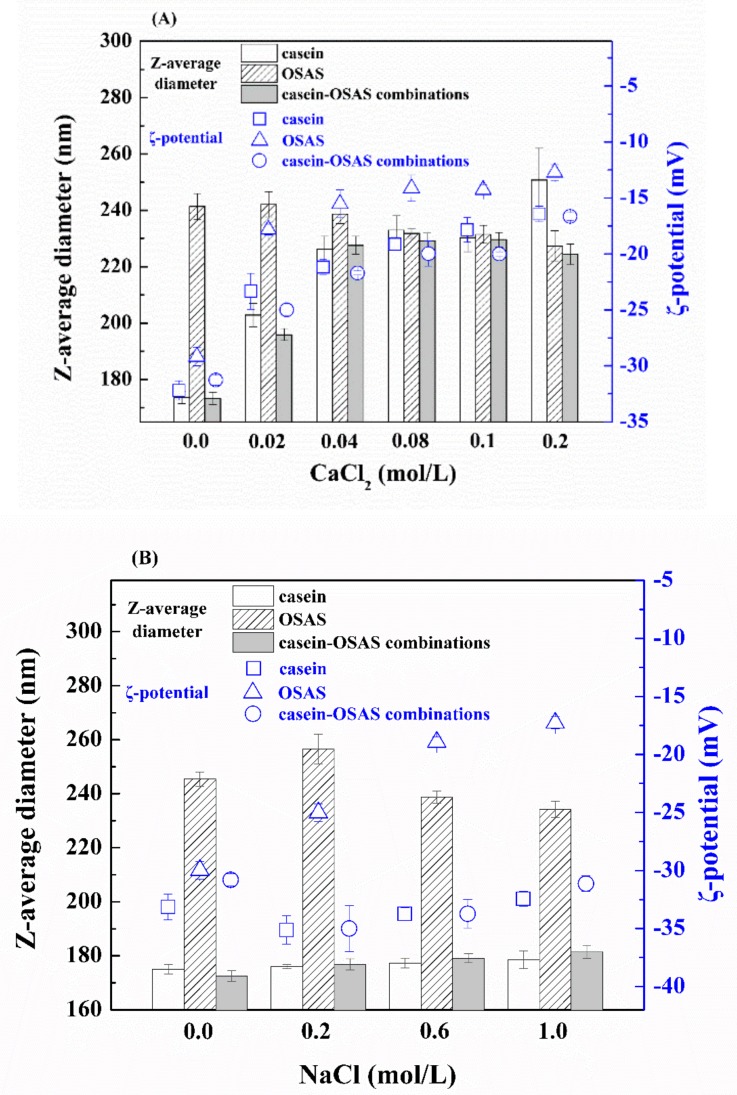
Influence of CaCl_2_ (**A**) and NaCl (**B**) concentration on the z-average diameter and ζ-potential of three emulsions stabilized by casein, OSAS, and the combination of casein and OSAS in a weight ratio of 8:2, respectively.

Commercial emulsion-based food systems often contain different concentrations of electrolytes in the aqueous phase. Hence, the influence of ionic strength on emulsion stability was examined (Figure 6). Compared with NaCl addition (Figure 6B), the addition of CaCl_2_ had a more profound effect on the z-average diameter and ζ-potential of the emulsions (Figure 6A). The z-average diameters of casein-stabilized nanoemulsions significantly increased by approximately 77 nm with increasing CaCl_2_ concentration from 0 to 0.2 mol/L (*p* < 0.05). This effect may be attributed to electrostatic screening and ion-binding effects [38], as confirmed by a significant decrease in the absolute value of ζ-potential (*p* < 0.05) (Figure 6A). Interestingly, there was an initial increase in z-average diameter in the emulsions prepared with the combination of casein and OSAS in a weight ratio of 8:2 or OSAS, followed by a decrease with increasing salts concentration. The z-average diameter in the OSAS-stabilized emulsion significantly decreased by 15 nm with increasing CaCl_2_ concentration from 0.02 to 0.2 mol/L (*p* < 0.05) while that in the nanoemulsion prepared with the combination of casein and OSAS in a weight ratio of 8:2 significantly decreased by 5 nm with increasing CaCl_2_ concentration from 0.1 to 0.2 mol/L (*p* < 0.05). Xu, Luo, Liu, and McClements also found a decrease in average particle size in emulsions containing xanthan gum with increasing NaCl level from 0 to 0.1 mol/L [39]. The possible reasons for this decrease are associated with changes in the strength of electrostatic interactions in the presence of a certain amount of salt. For example, a certain amount of salt in the emulsions will have weakened intramolecular electrostatic repulsion between the negatively charged groups (–COO^−^) on the OSAS molecules, thereby the chains could be packed closer together [39,40].

#### 3.2.4. Influence of Storage Conditions

**Figure 7:**
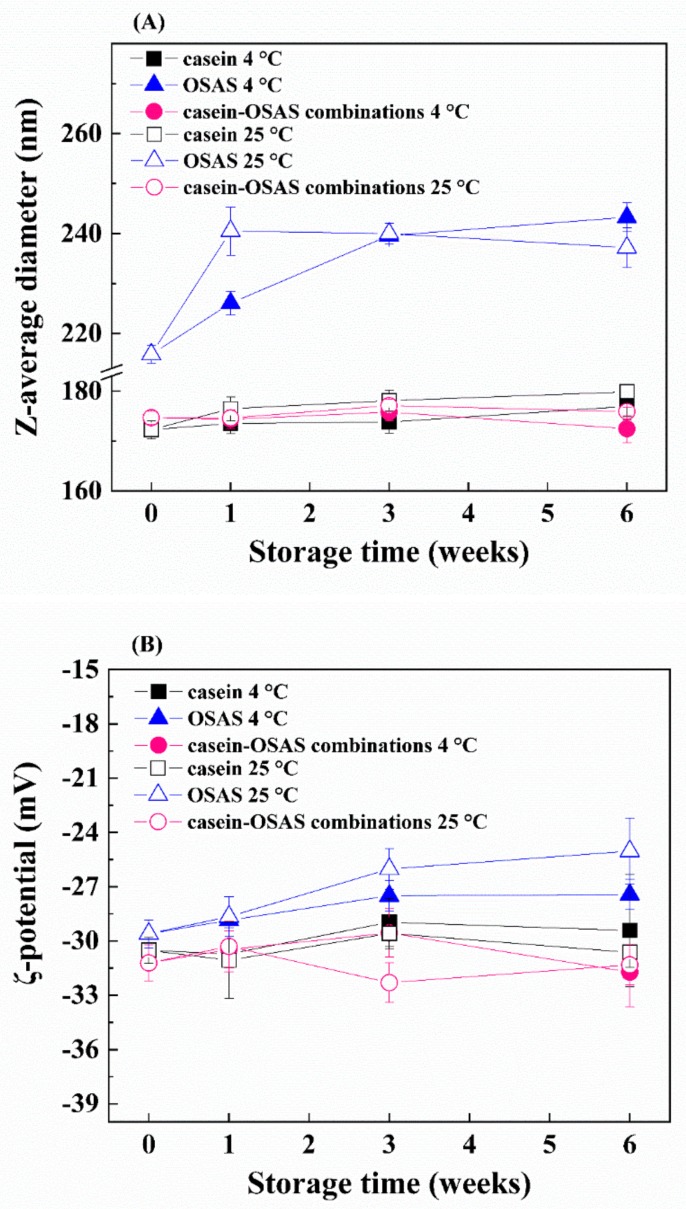

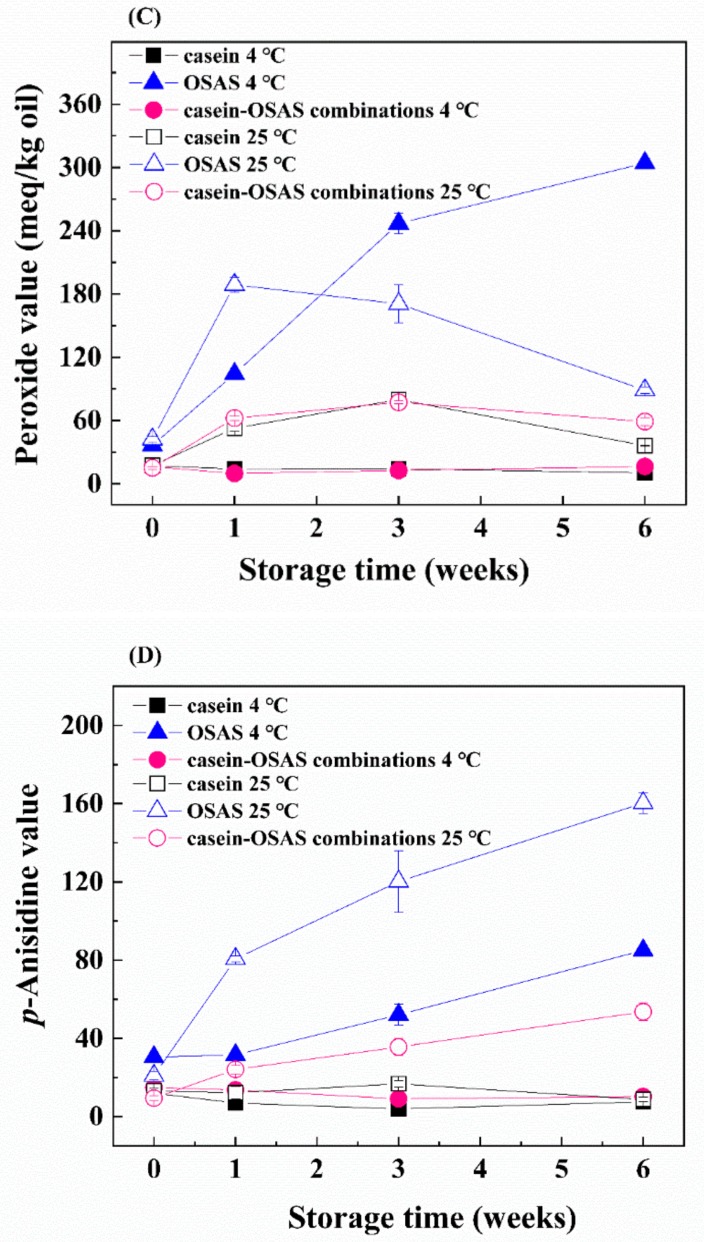
The z-average diameter (**A**), ζ-potential (**B**), peroxide value (**C**) and *p*-anisidine value (**D**) of three emulsions (prepared with casein, OSAS, and the combination of casein and OSAS in a weight ratio of 8:2, respectively) stored at different temperatures for 6 weeks.

Changes in the z-average diameter, ζ-potential, peroxide and *p*-anisidine values of emulsions were measured to evaluate the influence of storage conditions on the stability of the three emulsions prepared with casein, OSAS, and the combinations of casein and OSAS in a weight ratio of 8:2, respectively. Z-average diameters of nanoemulsions prepared with casein or the combination of casein and OSAS in a weight ratio of 8:2 stored at a higher temperature (25 °C) for 6 weeks became slightly larger than those stored at a lower temperature (4 °C) (Figure 7A). This phenomenon may be because the viscosity of the nanoemulsions at the low temperature decreased the droplet mobility, thereby leading to an attenuated droplet growth [41]. The largest increase in z-average diameter was observed in OSAS-stabilized emulsions stored at 4 and 25 °C for 6 weeks (Figure 7A). Presumably, some droplet aggregation in OSAS-stabilized emulsions occurred due to the reduced electrostatic repulsion between the droplets, which is supported by the ζ-potential measurement showing the absolute value of ζ-potential of the OSAS-stabilized emulsion was slightly lower than that of the two nanoemulsions (Figure 7B).

The peroxide value of OSAS-stabilized emulsions stored at 4 °C increased dramatically up to 6 weeks (*p* < 0.05), and it was significantly higher after 3 weeks than that of the emulsion stored at 25 °C (Figure 7C). Interestingly, the peroxide value of OSAS-stabilized emulsions stored at 25 °C significantly increased to 188.8 meq/kg oil at week 1 and decreased significantly after 1 week (*p* < 0.05). The decreased peroxide value suggested the decomposition rate of hydroperoxides in the emulsion stored at 25 °C was greater than the formation rate of hydroperoxides, compared with the emulsion stored at 4 °C. Presumably the secondary oxidation products formed may be higher. This phenomenon was supported by its *p*-anisidine value measurements, which shows the *p*-anisidine value of the OSAS-stabilized emulsion stored at 25 °C was markedly higher than that stored at 4 °C during the entire storage period (Figure 7D). Higher *p*-anisidine values representing greater formation of aldehydes may in turn cause a significant decrease in the absolute value of ζ-potential of the OSAS-stabilized emulsion after storing for 6 weeks (*p* < 0.05) (Figure 7B). This may be because the generated aldehydes further convert into acidic compounds during an oxidation process, resulting in a reduction in pH [42,43]. However, the oxidative stability of emulsions was greatly improved using the combination of casein and OSAS in a weight ratio of 8:2 as emulsifiers compared with using OSAS alone. Interestingly, Shi et al. reported that a thicker interfacial layer would provide steric hindrance to inhibit free radicals in the initial oxidation stage [44], while OSAS forming a thicker interfacial layer did not show the ability to protect lipids from oxidation (Figure 4B and Figure 7C–D). The reason for this may be that the interfacial layer formed by OSAS is thick but not dense enough to inhabit small molecule pro-oxidants from interacting with oil droplets. Moreover, although the interfacial layer containing casein is relatively thin, it could effectively prevent lipid oxidation due to the nature of casein, which acts as a natural antioxidant to inhibit lipid oxidation in nanoemulsions by scavenging free radicals and chelating transition metals [45,46,47]. Thus, a number of complex factors such as the types of emulsifiers and interfacial layer thickness affect the oxidative stability of lipids in the emulsion system. Further study is needed to explore the relationship between interfacial layer and lipid oxidation.

#### 3.2.5. Tribological Behaviors

**Figure 8:**
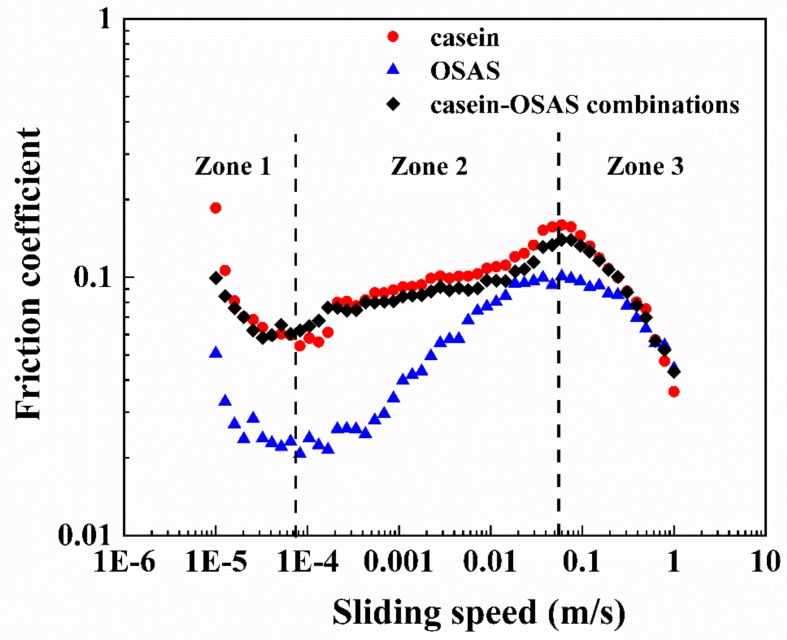
Schematic diagram of friction curves for three emulsions stabilized by casein, OSAS, and the combination of casein and OSAS in a weight ratio of 8:2, respectively.

The Stribeck curve is a classic friction model widely used in the field of lubrication friction. There are three regimes (boundary, mixed, and hydrodynamic lubrication) in the conventional Stribeck curve [48]. However, friction curves for the emulsion systems did not totally resemble the conventional Stribeck curve, as shown in Figure 8. Zone 1 and 2 resemble mixed and hydrodynamic lubrication in the conventional Stribeck curve, respectively. As the sliding speed increased, in zone 3, the emulsion structure may have been destroyed by the excessively high sliding speed [49]. The emulsion in the gap between the ball and plates might show the separation of oil and water phases. The appearance of the oil contributed to the formation and stability of the lubricating film, so the friction coefficient decreased. The tribological behavior in this zone is mainly affected by the structural strength of the emulsion [49,50]. The friction coefficient of the OSAS-stabilized emulsion was significantly smaller than that of the two nanoemulsions, while the friction curve of the nanoemulsion stabilized by the combination of casein and OSAS in a weight ratio of 8:2 almost coincided with that of the casein-stabilized nanoemulsion. However, the friction curve of the OSAS- stabilized emulsion was converted from zone 2 to zone 3 at a relatively low sliding speed compared with the other two curves, indicating the emulsion stabilizing by OSAS alone was relatively weak against high-speed mechanical strength. The tribological results showed that emulsions formed by different emulsifiers had different tribological behaviors and further study is needed to understand the relationship between the structure and tribological properties of O/W emulsions. Since mechanical properties of food often affect sensory texture, the results of the study indicated that it is of vital importance to design emulsions with specific tribological behaviors by controlling the emulsifier types of emulsions.

## 4. Conclusions

The nanoemulsion stability to environment stresses could be greatly improved by using casein and OSAS emulsifier combinations. In particular, droplet aggregation around the isoelectric point of casein could be dramatically inhibited by adding OSAS. This effect was attributed to the formation of casein–OSAS combinations to increase steric and electrostatic repulsion between the droplets. There was also an interaction between casein and OSAS via hydrogen bonding as evidenced by FTIR. Alternatively, nanoemulsions stabilized by casein–OSAS combinations or casein showed better oxidative stability than OSAS-stabilized emulsions. Tribological behavior of emulsions indicated the possibility of designing emulsions with specific textures by controlling the emulsifier types of emulsions. Some physicochemical stability of emulsions could be improved by casein–OSAS combinations as emulsifiers compared with each of the two emulsifiers alone, which shows potential for wide applications of protein-modified starch combinations in emulsion-based products.

## Figures and Tables

**Table 1:** The single-factor tests for nanoemulsions preparation.

Factors	Casein/OSAS Weight Ratio (Total Mass Fraction of 7%)	MCTs/Fish Oil Weight Ratio	Oil Phase Concentration	Ultrasonic Time (min) *	Ultrasonic Power (W)
Casein/OSAS weight ratio	0:10, 2:8, 5:5, 8:2, 10:0	5:5	4%	6	480
MCTs/fish oil weight ratio	8:2	0:10, 2:8, 5:5, 7:3, 8:2	4%	6	480
Oil phase concentration	8:2	0:10	1%, 2%, 3%, 4%, 5%	6	480
Ultrasonic time	8:2	0:10	1%	2, 6, 10, 14, 18, 22	480
Ultrasonic power	8:2	0:10	1%	22	120, 240, 360, 480, 600

* The ultrasonic time was total processing time including the pulsing times.
